# Incorporation of enzyme concentrations into FBA and identification of optimal metabolic pathways

**DOI:** 10.1186/1752-0509-2-65

**Published:** 2008-07-18

**Authors:** Rajat K De, Mouli Das, Subhasis Mukhopadhyay

**Affiliations:** 1 Machine Intelligence Unit, Indian Statistical Institute, Kolkata 700108, India; 2 Department of Biophysics, Molecular Biology and Genetics, Calcutta University, Kolkata 700009, India

## Abstract

**Background:**

In the present article, we propose a method for determining optimal metabolic pathways in terms of the level of concentration of the enzymes catalyzing various reactions in the entire metabolic network. The method, first of all, generates data on reaction fluxes in a pathway based on steady state condition. A set of constraints is formulated incorporating weighting coefficients corresponding to concentration of enzymes catalyzing reactions in the pathway. Finally, the rate of yield of the target metabolite, starting with a given substrate, is maximized in order to identify an optimal pathway through these weighting coefficients.

**Results:**

The effectiveness of the present method is demonstrated on two synthetic systems existing in the literature, two pentose phosphate, two glycolytic pathways, core carbon metabolism and a large network of carotenoid biosynthesis pathway of various organisms belonging to different phylogeny. A comparative study with the existing extreme pathway analysis also forms a part of this investigation. Biological relevance and validation of the results are provided. Finally, the impact of the method on metabolic engineering is explained with a few examples.

**Conclusions:**

The method may be viewed as determining an optimal set of enzymes that is required to get an optimal metabolic pathway. Although it is a simple one, it has been able to identify a carotenoid biosynthesis pathway and the optimal pathway of core carbon metabolic network that is closer to some earlier investigations than that obtained by the extreme pathway analysis. Moreover, the present method has identified correctly optimal pathways for pentose phosphate and glycolytic pathways. It has been mentioned using some examples how the method can suitably be used in the context of metabolic engineering.

## Background

Metabolism is a complex process that takes place for producing energy and forms the driving force for cellular activity. It involves a large number of chemical reactions/conversions carried out by living organisms as they feed, grow and reproduce. A cascade of such reactions/conversions form a highly branched network. A metabolic network consists of many reactions and transport processes associated with the production and depletion of cellular metabolites. Metabolic pathways are defined as coordinated series of biochemical reactions in which the product of one reaction is the reactant of the subsequent one in the chain. Examples of metabolic pathways include Glycolysis, the Krebs cycle and the Pentose phosphate pathways.

There exist various categories of data models for analyzing metabolic pathways. The huge amount of genomic data, available at present, has led to the construction of genome-scale models of metabolism [1]. The biological information from genomes can be extracted by constructing computational models and subsequently making predictions from them [2,3]. Flux balance analysis is a constraint-based approach [4-6] that spans the closed solution space within which many steady state solutions would lie. Optimization techniques are used to find out a single state, within this space of allowed states, which reflects the actual flux distribution of the cell under a defined set of nutrient conditions [7,8]. The utilities of such modeling include predicting systems behavior, identifying crucial steps in systems regulation. In [9], Cascante et. al. have shown how this kind of modeling can be used for characterizing fermentation pathway of *S. cerevisiae*. Moreover, modeling and analysis of metabolic networks may be useful to perform rational drug design [10].

Reactions in a metabolic pathway are mostly enzymatic. That is, for a reaction A → B catalyzed by an enzyme E, the rate of production of B depends not only on the concentration of the substrate A but also on the concentration of E that is available for catalyzing the reaction. Assuming that sufficient amount of the substrate A being present, if the concentration of E is low (high) then the rate of production of B will also be low (high). In the extreme pathway analysis (one of the methods under flux balance approach) [11], the authors have considered the reaction flux but not the enzyme concentration. This motivates us to develop a new method that considers both the substrate and enzyme concentration, thereby it becomes somewhat closer to real life situations than what extreme pathway analysis offers. We intend to undertake this endeavor in the present article.

**Figure 1:**
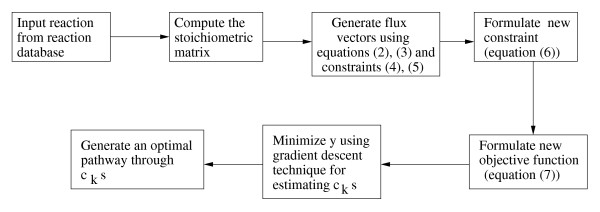
**Outline of the proposed method**. Outline of the proposed method.

Here we develop a method for identification of a metabolic pathway, in terms of the level of enzyme concentration, which yields the maximum rate of production of a metabolite in the pathway starting from a given substrate. The method determines an optimal set of enzymes that is required to get an optimal metabolic pathway through which the rate of production of a metabolite is maximum. In other words, the method is able to determine a set of enzymes that needs to be expressed at a certain level for increasing the production of the target metabolite. The method, first of all, generates the possible flux vectors in the pathway. For this purpose, assuming steady state condition, we consider the basis vectors that span the null space of the given stoichiometric matrix. Then we take convex combination of these basis vectors to generate the flux vectors that satisfy certain inequality constraints. A set of weighting coefficients, corresponding to enzymes catalyzing biochemical reactions in the said pathway, is incorporated, and then a set of constraints incorporating these weighting coefficients is formulated. An objective function, in terms of these weighting coefficients, is formed, and then minimized under regularization method. The weighting coefficients corresponding to a minimum value of the objective function represent an optimal pathway. Fig. 1 depicts the flowchart of the method that is easy to implement, yet workable. For simplicity, we have made some assumptions as mentioned in the methodology section.

The effectiveness of the present method is demonstrated on two synthetic systems designed in [12,13], on two pentose phosphate, two glycolytic pathways [14], one large carotenoid biosynthesis pathway [14] and a network of core carbon metabolism [15] of various organisms belonging to different phylogeny. The method is compared with the existing extreme pathway analysis [11]. The major differences of the present method from the existing extreme pathway analysis have been pointed out. Finally, we provide biological relevance of the results. A possible validation from biological point of view along with the salient features of the method is also included. It has been demonstrated with a few examples that the method can be appropriately applied to the problems of metabolic engineering.

## Results

The proposed method is described in the methodology section. Here we provide a comparative analysis of the present method with extreme pathway analysis using two synthetic [12,13] and four different real life pathways. Real life pathways include pentose phosphate and glycolytic pathways of *E. coli *K-12 MG1655, *T. pallidum *and *P. falciparum*, a large network of carotenoid biosynthesis [16-20] and a network of core carbon metabolism [15]. All these real life pathways are obtained from the KEGG database [14]. In order to restrict the size of the article, we have provided a brief account on these real-life pathways in the Additional File 1. Some of the results are included here while the others are provided in the Additional File 1 for restricting the size of the article.

### Analysis of the results

#### On the synthetic system in Fig. 2

**Figure 2:**
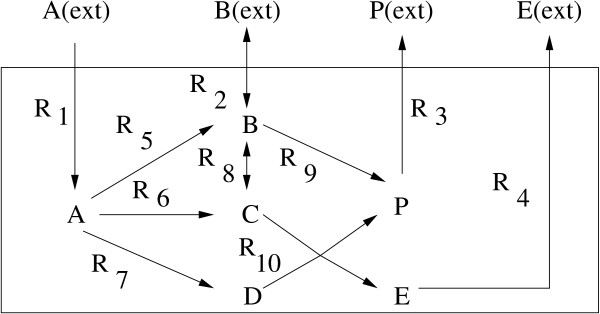
**Synthetic reaction system 1**. A chemical reaction network consisting of 6 metabolites and 10 reactions.

For the system in Fig. 2, we want to maximize the rate of yield of the metabolite P, starting from the substrate A. The system is composed of 10 reactions *R*_1_, *R*_2_,...,*R*_10 _involving 6 metabolites A, B, C, D, E and P. The reactions *R*_2 _and *R*_8 _are reversible. We associate the weighting factors *c*_1_, *c*_2_,...,*c*_10 _corresponding to the enzymes catalyzing these reactions respectively. There are 6 internal fluxes *R*_5_, *R*_6_,...,*R*_10 _and 4 exchange fluxes *R*_1_, *R*_2_,...,*R*_4 _as depicted in Fig. 2. The constraints as mentioned in the methodology section, and following [13] are as follows:

*α *= (0, -∞, 0, 0, 0, 0, 0, -∞, 0, 0); *β *= (1, 0, ∞, ∞, ∞, ∞, ∞, ∞, ∞, ∞).

The terms *α*_2 _and *α*_8 _are -∞ because *R*_2 _and *R*_8 _are reversible. Moreover, *β*_2 _= 0 because exclusive growth on substrate A is considered. Finally, we assume that the maximal uptake rate of A is 1 (*β*_1 _= 1). Following the method described in the methodology section, we have generated a set of flux vectors.

In order to maximize the rate of yield of P for growth on substrate A, the objective function *y *(Eq. (7)) is minimized, where *z *is given by *z *= *c*_9_*v*_9 _+ *c*_10_*v*_10 _- *c*_3_*v*_3_. We vary the value of *λ *from 0.1 to 1.0. Initially, we should always give stress on the maximization of the rate of yield rather than on the constraint. That is, initially *λ *should be kept small. As we go from *λ *= 0.1 to *λ *= 1.0, it implies that we are increasing the stress on the constraint, and finally both the rate of yield (*z*) and the constraint are treated equally. For each value of *λ*, we minimize *y*, and consider that set of *c*_*i*_-values corresponding to the *λ*-value as the final solution, for which *y *becomes minimum. Here we have obtained an optimal pathway as *R*_1 _→ *R*_5 _→ *R*_9 _→ *R*_3_, which is in accordance with earlier investigations [13]. The optimal pathway is obtained for *λ *= 1.0 in 85 iterations.

Table 1 shows a few pathways along with *c*-values and average amount (*z*) of the target P. Since, we have generated a set of flux vectors, we have considered average of these vectors to compute the average amount of the target product P. For example, the pathway *R*_1 _→ *R*_5 _→ *R*_9 _→ *R*_3 _yields the highest average *z*, and hence it is the optimal pathway. It is to be mentioned here that the paths involving the reactions *R*_6 _and *R*_7 _need to be activated to yield C and D respectively, as both C and D are required to produce P through these paths. The other synthetic pathway is included in Fig. 8 in Additional File 1 in order to restrict the size of the article.

**Table 1:** Some possible pathways for the system in Fig. 2 (or Fig. 6)

Serial Number	Some possible paths	Optimal c-values	Average quantity (z) of P
1	*R*_1 _→ *R*_5 _→ *R*_9 _→ *R*_3_	*c*_1 _= 0.88, *c*_5 _= 0.80, *c*_9 _= 0.80, *c*_3 _= 0.86	51.53
2	*R*_1 _→ *R*_6 _→ *R*_8 _→ *R*_9 _→ *R*_3_	*c*_1 _= 0.88, *c*_6 _= 0.56, *c*_8 _= 0.57, *c*_9 _= 0.80, *c*_3 _= 0.86	12.22
3	*R*_1 _→ *R*_5 _→ *R*_8 _→ *R*_10 _→ *R*_3_	*c*_1 _= 0.88, *c*_5 _= 0.80, *c*_8 _= 0.57, *c*_10 _= 0.04, *c*_3 _= 0.86	24.63
4	*R*_1 _→ *R*_6 _→ *R*_10 _→ *R*_3_	*c*_1 _= 0.88, *c*_6 _= 0.56, *c*_10 _= 0.04, *c*_3 _= 0.86	19.88
5	*R*_1 _→ *R*_7 _→ *R*_10 _→ *R*_3_	*c*_1 _= 0.88, *c*_7 _= 0.18, *c*_10 _= 0.04, *c*_3 _= 0.86	29.41

We have varied the upper bound of the flux values to show the variation of enzyme concentrations (*c*-value) and the amount (*z*) of the target metabolite. The results are provided in Table 2 for some high and low upper bounds. It is clear from Table 2 that *z*-value, as expected, decreases with the decrease in upper bound. In all the cases, we have found the same optimal path although absolute *c*-values differ. This shows the consistency of the proposed method in determining optimal metabolic paths.

**Table 2:** Variation of c-values and average z with the upper bound on reaction fluxes for the optimal path *R*_1 _→ *R*_5 _→ *R*_9 _→ *R*_3 _of the system in Fig. 2

Serial Number	Upper bound on flux value	Optimal c-values	Average quantity (z) of P
1	5000	*c*_1 _= 0.93, *c*_5 _= 0.83, *c*_9 _= 0.91, *c*_3 _= 0.85	6670.68
2	4000	*c*_1 _= 0.91, *c5 *= 0.92, *c*_9 _= 0.82, *c*_3 _= 0.98	5458.83
3	3000	*c*_1 _= 0.91, *c*_5 _= 0.85, *c*_9 _= 0.83, *c*_3 _= 0.91	4308.66
4	2000	*c*_1 _= 0.88, *c*_5 _= 0.84, *c*_9 _= 0.81, *c*_3 _= 0.86	3451.73
5	1000	*c*_1 _= 0.87, *c*_5 _= 0.82, *c*_9 _= 0.86, *c*_3 _= 0.83	2347.61
6	50	*c*_1 _= 0.84, *c*_5 _= 0.89, *c*_9 _= 0.87, *c*_3 _= 0.82	55.69
7	40	*c*_1 _= 0.94, *c*_5 _= 0.87, *c*_9 _= 0.89, *c*_3 _= 0.81	47.29
8	30	*c*_1 _= 0.89, *c*_5 _= 0.86, *c*_9 _= 0.85, *c*_3 _= 0.85	42.57
9	20	*c*_1 _= 0.86, *c*_5 _= 0.84, *c*_9 _= 0.81, *c*_3 _= 0.82	38.66
10	10	*c*_1 _= 0.98, *c*_5 _= 0.93, *c*_9 _= 0.91, *c*_3 _= 0.87	34.96

#### On the Glycolytic Pathway in *T. pallidum *(Fig. 3)

**Figure 3:**
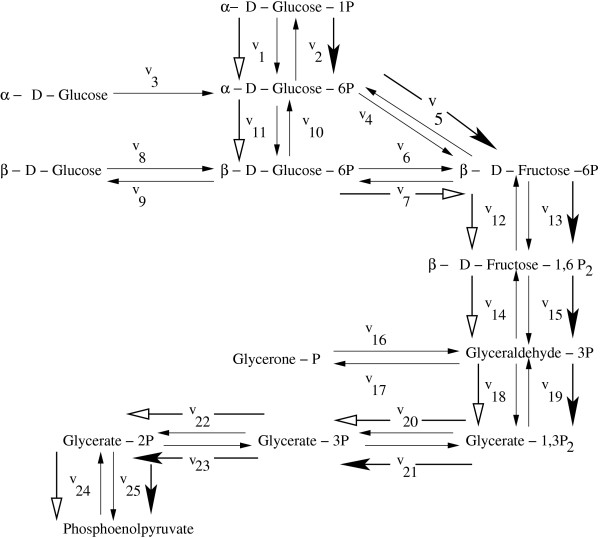
**Glycolytic Pathway in *T. pallidum***. Glycolytic pathway in *T. pallidum *consisting of 13 metabolites and 25 fluxes (reversible reactions are shown by double arrows). The starting metabolite is *α*-D-Glucose-1P and the target product is phosphoenolpyruvate respectively. The bold (black) arrows represent the optimal pathway obtained by the present method and the bold (white) arrows represent the optimal pathway obtained by the extreme pathway analysis.

The glycolytic pathway in *T. pallidum *consists of 13 metabolites and 25 fluxes (Fig. 3). The starting metabolite is *α *-D-Glucose-1P and the target product is phosphoenolpyruvate. Thus we maximize the rate of yield *z *= *c*_25_*v*_25 _- *c*_24_*v*_24 _of phosphoenolpyruvate, starting from the substrate *α*-D-Glucose-1P. Here an optimal pathway has been obtained as *α *- *D *- *Gluucose *- 1*P *→ *α *- *D *- *Gluucose *- 6*P *→ *β *- *D *- *Fructose *- 6*P *→ *β *- *D *- *Fructose *- 1, 6*P*2 → *Glyceraldehyde *- 3*P *→ *Glycerate *- 1, 3*P*2 → *Glycerate *- 3*P *→ *Glycerate *- 2*P *→ *Phosphoenolpyruvate *in 100 iterations as shown by bold (black) arrows. The optimal pathway is obtained for *λ *= 0.9.

#### On the Carotenoid biosynthesis pathway (Fig. 9 in Additional File 1)

**Figure 4:**
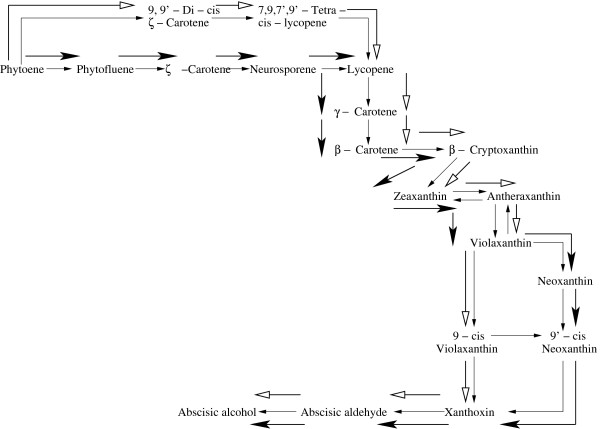
**Optimal Carotenoid biosynthesis Pathway**. The bold (black) arrows represent the optimal pathway obtained by the present method and the bold (white) arrows represent that found by the extreme pathway analysis.

Considering the reference pathway from KEGG database, the starting metabolite for the carotenoid biosynthesis pathway is phytoene and the target metabolite is abscisic alcohol [21,22]. There are 83 metabolites and 100 fluxes (Fig. 9 in Additional File 1). There are 2 reversible and 98 irreversible reactions. Applying the present methodology, optimal pathway for the carotenoid biosynthesis has been found to be: *Phytoene *→ *Phytofluene *→ *ζ *- *Carotene *→ *Neurosporene *→ *Lycopene *→ *γ *- *Carotene *→ *β *- *Carotene *→ *β *- *Cryptoxanthin *→ *Zeaxanthin *→ *Antheraxanthin *→ *V iolaxanthin *→ *Neoxanthin *→ 9' - *cis *- *Neoxanthin *→ *Xanthoxin *→ *Abscisic aldehyde *→ *Abscisic alcohol*. The optimal pathway is obtained for *λ *= 0.7 in 90 iterations, which is shown in Fig. 4 by bold (black) arrows.

#### On the core carbon metabolic network (Fig. 5)

**Figure 5:**
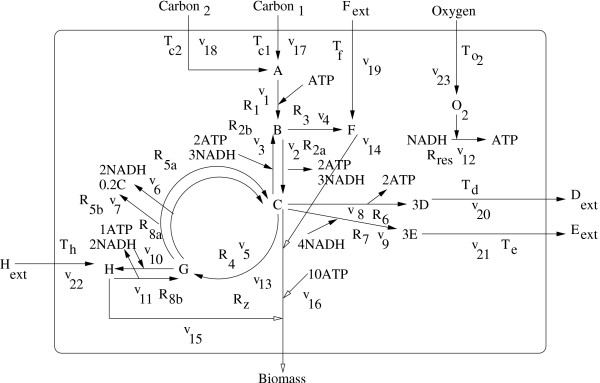
**Core carbon metabolic network**. A simplified core carbon metabolic network [15]. The network consists of 12 metabolites and 23 reactions. The stoichiometry of the metabolic reactions are described as follows [15]: *R*_1 _: *A *+ *ATP *→ *B*; *R*_2*a *_: *B *→ 2*ATP *+ 2*NADH *+ *C*; *R*_2*b *_: *C *+ 2*ATP *+ 2*NADH → B*; *R*_3 _: *B *→ *F *; *R*_4 _:*C *→ *G*; *R*_5*a *_: *G *→ 0.8*C *+ 2*NADH*; *R*_5*b *_: *G *→ 0.8*C *+ 2*NADH*; *R*_6 _: *C *→ 2*ATP *+ 3*D*; *R*_7 _: *C *+ 4*NADH *→ 3*E*; *R*_8*a *_: *G *+ *ATP *+ 2*NADH *→ *H*; *R*_8*b *_: *H *→ *G *+ *ATP *+ 2*NADH*; *R*_*res *_:*NADH *+ *O*_2 _→ *ATP*; *C *→ *Biomass*; *F *→ *Biomass*; *H *→ *Biomass*; 10*ATP *→ *Biomass*. The stoichiometry of the transport processes are described as follows: *T*_*c*1 _: *Carbon*1 → *A*; *T*_*c*2 _: *Carbon*2 → *A*; *T*_*f *_: *Fext *→ *F*; *T*_*d *_: *D *→ *Dext*; *T*_*e*_: *E *→ *Eext*; *T*_*h *_: *Hext *→ *H*; *T*_*o*2 _: *Oxygen *→ *O*2. The biomass flux *R*_*z *_is as follows: *C *+ *F *+ *H *+ 10*ATP *→ 1 *Biomass *is indicated by white arrows.

We have applied the method to analyze a simple example that represents the skeleton network of core carbon metabolism [15,23]. The network includes 23 reactions, seven of which are regulated by four regulatory proteins. The internal metabolites are A, B, C, D, E, F, O_2_, NADH, ATP and the external metabolites are Carbon 1, Carbon 2, *D*_*ext*_, *E*_*ext*_, *F*_*ext*_, *H*_*ext *_and Oxygen. This network is a highly simplified representation of core metabolic processes including a glycolytic pathway with carbon 1 (C1) and carbon 2 (C2) as primary substrates, as well as a pentose phosphate pathway and a TCA cycle, through which amino acid H enters into the system. Fermentation pathways, amino acid biosynthesis, cell growth along with corresponding regulation (e.g. catabolite repression, aerobic/anaerobic regulation, and carbon storage regulation) are also included. The growth reaction is indicated by white arrows. For further details of the pathway, one may refer to [15,23].

Our methodology aims at obtaining the optimal metabolic flux distribution within the solution space. In this study, *R*_*z*_, the biomass flux, is defined as *C *+ *F *+ *H *+ 10*AT P *→ 1 *Biomass*, and needs to be maximized. Applying the proposed methodology, the optimal pathway has been found to be: *A *→ *B *→ *C *→ *G *→ *C *→ *Biomass *for *λ *= 0.8 in 95 iterations.

### Comparison with the extreme pathway analysis [11,24]

Extreme pathways are a set of generating vectors that describe the conical steady-state solution space for flux distributions through an entire metabolic network. These cone-generating vectors correspond to biochemical pathways. The optimal metabolic pathways are calculated using linear optimization and are then interpreted using the extreme pathways. For details of the method, one may refer to [11,24]. Here we demonstrate a comparative analysis of the present method with the extreme pathway analysis [11,24]. The comparative analysis has been done on all the above mentioned pathways. Optimal pathways obtained by extreme pathway analysis for the two synthetic systems (Fig. 2, and Fig. 8 in Additional File 1) are the same as that obtained by the present method. Similarly, for pentose phosphate and glycolytic pathways in *E. coli *K-12MG1655 (Figs. 10 and 11 in Additional File 1), optimal pathways are the same as obtained by both the methods.

For *P. falciparum*, an optimal pentose phosphate pathway *α *- *D *- *Glucose *- 6*P *→ *β *- *D *- *Fructose *- 6*P *→ *D *- *Xylulose *- 5*P *→ *D *- *Glyceraldehyde *- 3*P *as obtained by the extreme pathway analysis was found to be different from that obtained by the present method as shown in Fig. 12 in Additional File 1. The glycolytic pathway in the organism *T. pallidum *as obtained by the present algorithm has been found to be different from the previously identified optimal pathway by the extreme pathway analysis. For the latter case, it was found to be *α *- *D *- *Glucose *- 1*P *→ *α *- *D *- *Glucose *- 6*P *→ *β *- *D *- *Glucose *- 6*P *→ *β *- *D *- *Fructose *- 6*P *→ *β *- *D *- *Fructose *- 1, 6*P*2 → *Glyceraldehyde *- 3*P *→ *Glycerate *- 1, 3*P*2 → *Glycerate *- 3*P *→ *Glycerate *- 2*P *→ *Phosphoenolpyruvate *as shown by bold (white) arrows in Fig. 3. Using the extreme pathway analysis we have obtained a different carotenoid biosynthesis pathway: *Phytoene *→ 9, 9' - *Di *- *cis *- *ζ *- *Carotene *→ 7, 9, 7', 9' - *Tetra *- *cis *- *lycopene *→ Lycopene → *γ *- *Carotene *→ *β *- *Carotene *→ *β *- *Cryptoxanthin *→ *Zeaxanthin *→ *Antheraxanthin *→ *Violaxanthin *→ 9 - *cis *- *Violaxanthin *→ *Xanthoxin *→ *Abscisic aldehyde *→ *Abscisic alcohol *as shown by bold (white) arrows in Fig. 4.

For the carotenoid biosynthesis pathway, the path obtained by the proposed method is different from the extreme pathway analysis at the starting branch and at the end of the branch of the optimal path.

Starting from Phytoene there are two paths to arrive at the intermediate metabolite Lycopene. Of the two paths, the proposed method found three metabolites, Phytofluene, *ζ*-Carotene and Neurosporene while the extreme pathway analysis identified two metabolites, 9,9'-Di-cis-*ζ*-Carotene and 7,9,7',9'-Tetra-cis-lycopene, to reach Lycopene, as can be seen from Fig. 4. The paths identified by the above two methods are different till it reaches the metabolite Lycopene. From Lycopene both the methods follow the same path till they arrive at the other intermediate metabolite Violaxanthin. From Violaxanthin extreme pathway method found one intermediate metabolite, 9-cis-Violaxanthin to arrive at the other intermediate metabolite Xanthoxin. The proposed method found two intermediate metabolites Neoxanthin and 9'-cis-Neoxanthin to reach Xanthoxin. So we can conclude that from Violaxanthin the paths obtained by the above two methods are differnt till it arrives at the metabolite Xanthoxin. From Xanthoxin both the methods follow the same path to reach the target metabolite Abscisic alcohol.

Each extreme pathway of core carbon metabolism was scaled to its maximum possible flux based on the maximum value of the uptake reactions (*v*_*max*_) given in [15]. Here we have assumed that there is no restriction on the environmental conditions and all possible inputs are available. The environment contains carbon1 (C1), F, H, *O*_2 _and the transport flux *T*_*c*2 _is repressed in the presence of C1. We have not taken into account the regulatory constraints associated with regulation of gene expression, i.e., by repressing or activating certain genes and other environmental conditions.. The regulatory and environmental constraints may further constrain the allowable functions of the network. The pathway obtained by the proposed method is similar to pathway 32 as obtained by the extreme pathway analysis in [23]. The article also derives two sets of optimal pathways in terms of the highest biomass yield with no byproduct secretion. The optimal pathway with the highest yield obtained by our method is similar to pathway 32 of group 1 [23]. A comparison of the flux values obtained from our methodology with the extreme pathway analysis and their percentage deviations are demonstrated in Table 3. From the table it can be inferred that the flux values obtained by both methods are more or less similar in nature although some external flux values deviate considerably. These considerable deviations may be due to the following reasons.

**Table 3:** Comparison of the flux values obtained by the proposed method and the extreme pathway analysis for the core carbon metabolic network in Fig. 5

Reaction	*v*_*max*_	*v*_*av *_	Percentage deviation
	(Extreme path- way analysis)	(Proposed method)	|*v*_*av *_- *v*_*max*_|/*v*_*av *_× 100%
Tc1	10.5	11.13	5.66
Tc2	10.5	11.43	8.13
Td	12	13.91	13.73
Te	12	10.47	14.61
Tf	5	7.65	34.64
Th	5	6.84	26.90
To2	15	12.63	18.76

The values of *v*_*max *_in Table 3 corresponding to extreme pathway analysis were obtained by imposing certain environmental and regulatory constraints mentioned in [23], while the proposed method, for simplicity, does not consider such constraints. Moreover, for computing an average flux value by our method, we have taken average of a distribution of such flux values while the method of extreme pathway analysis determines the flux value. Finally, it may be mentioned here that we have developed the methodology accommodating certain characteristics of a system (i.e. within a specific metabolic system).

We did not consider the characteristics outside the system, from which the external fluxes enter into it. However, the method developed here has produced consistent results that have been validated by randomizing the starting point in generating flux vectors.

We have compared the flux values obtained by the proposed method with that derived by extreme pathway analysis. The results are shown for the system in Fig. 2 and for the core carbon metabolic network in Fig. 5. Since the proposed method, unlike extreme pathway analysis, generates a number of flux values corresponding to a single reaction, we have taken average of these values for the reaction and used this average for comparison. Percentage deviations between average flux values (*v*_*av*_) and flux values (*v*_*epa*_) derived by extreme pathway analysis were calculated in Table 4. It is clear from the table that the flux values corresponding to both these methods are very close; although, as in the case of Table 3, some considerable deviations were noted mostly for external fluxes. The reasons for such deviations for the carbon metabolic pathway (Fig. 5) have been explained in the paragraph above. Note that here no constraint was considered by both the methods.

**Table 4:** Comparison of flux values obtained by the proposed method and the extreme pathway analysis for the system in Fig. 2

Reaction	Average flux value (proposed method)	Flux value (Extreme pathway analysis)	Percentage deviation
	(*v*_*av*_)	(*v*_*epa*_)	|*v*_*av *_- *v*_*epa*_|/*v*_*av *_× 100%
R1	48.73	46.21	5.17
R2	3.596	3.129	12.98
R3	36.286	32.543	10.31
R4	7.687	6.292	18.15
R5	49.227	46.341	5.86
R6	17.86	16.001	10.41
R7	12.35	12.31	0.32
R8	14.50	13.656	5.82
R9	68.318	65.734	3.78
R10	15.263	14.814	2.94

### Biological relevance and validation

This section provides how the results obtained by the present method as well as extreme pathway analysis are relevant to some biological facts already observed by other researchers. Salient features of the present method along with true/false positive/negative scenarios are also depicted.

#### Relevance

Here we demonstrate how the results obtained by the present method are biologically more relevant than those obtained by the extreme pathway analysis.

In the carotenoid biosynthesis pathway, there are two possible paths from the initial metabolite phytoene, producing 9,9'-Di-cis-*ζ*-Carotene in one branch and phytofluene in another branch. Of the two possible paths, the branch that produces phytofluene as the intermediate metabolite is observed in [25,26], which is the same as obtained by our proposed method (Fig. 4). It is to be mentioned here that the other path has been identified by the extreme pathway analysis.

As we proceed along the path, we observe that there are 4 possible paths emerging from the intermediate metabolite Neurosporene (Fig. 9 in Additional File 1). The path that produces *α*-Zeacarotene and Hydroxy-neurosporene are not biochemically feasible as they do not lead to the target metabolite Abscisic alcohol. Of the remaining two paths, the path producing Lycopene is obtained by the present method (Fig. 4). The path that leads from phytoene to lycopene through the intermediate paths as mentioned above can be found in fungi [27,28]. Lycopene is also found to be an intermediate in the biosynthesis of other carotenoids, in some bacteria, fungi and green plants [29]. Thus both the present and extreme pathway analysis have correctly identified Lycopene as an intermediate over the other alternative *β*-Zeacarotene (Fig. 4).

There are 4 possible paths emerging from the intermediate metabolite Lycopene (Fig. 9 in Additional File 1). The paths producing *δ*-Carotene, 3,4-Dehydrolycopene and Rhodopin as the intermediate metabolites are not possible as they do not lead to the final product Abscisic alcohol. We can reach the target metabolite Abscisic alcohol only through the path that produces *γ*-carotene. There are 7 possible paths from the intermediate metabolite *γ*-carotene (Fig. 9 in Additional File 1). The paths yielding Chlorobactene, 1'-Hydroxy- *γ*-carotene, Myxol, Deoxymyxol, (2'S)-Deoxymyxol 2'-*α*-L- fucoside and 1'2'-Dihydro-*γ*-carotene do not terminate to the target metabolite Abscisic alcohol, and hence they are not biochemically feasible. The target metabolite can be obtained through the path producing *β*-carotene. The biosynthesis pathway for *β*-carotene has been determined for fungi such as Phycomyces blakesleeanus and Neurospora crassa [30]. *β*-carotene is also synthesized by a number of bacteria, fungi, and most green plants [30]. The path from *β*-carotene producing Echinenone terminates at the end products Adonixanthin and Astaxanthin. The other path terminates at the end product Isorenieratene. The above mentioned two paths are not possible as none of them yields Abscisic alcohol that is the desired target metabolite of carotenoid biosynthesis (Fig. 9 in Additional File 1). The path producing *β*-Cryptoxanthin is the only feasible path as it finally leads to the target metabolite.

From *β*-Cryptoxanthin there are two paths producing Thermo-biszeaxanthin and Zeaxanthin as the two products. The path producing Zeaxanthin is followed as it terminates to the desired end metabolite. The path from *β*-carotene to Zeaxanthin can be found in Flavobacterium Species [31]. The conversion between Zeaxanthin and Antheraxanthin is a reversible one, and as the forward reaction rate is greater than the reverse rate, the pathway from Zeaxanthin to Antheraxanthin is favored. As Capsanthin obtained from Antheraxanthin is not the desired end product (Fig. 9 in Additional File 1), this path is not followed. Violaxanthin obtained from Antheraxanthin via the reversible reaction ultimately leads to the target metabolite Abscisic alcohol. Capsorubin is obtained from Violaxanthin but this path is not followed as this does not yield the desired target metabolite Abscisic alcohol. Neoxanthin is produced from Violaxanthin. Arabidopsis is the best-characterized plant system of carotenoid biosynthesis. The path from *β*-carotene to Neoxanthin for xanthophyll biosynthesis has been observed in Arabidopsis. It may be emphasized that this path was identified by the present method but not by the extreme pathway analysis (Fig. 4).

Similarly, there exists a single path from Neoxanthin to 9'-cis-Neoxanthin and from 9'-cis-Neoxanthin to Xanthoxin. Two paths are emerging from Xanthoxin producing Abscisic aldehyde in one branch and Xanthoxic acid in the other. The path leading to Xanthoxic acid is not followed as this does not lead to the final metabolite of carotenoid biosynthetis. The other one producing Abscisic aldehyde is followed as it terminates to the target metabolite Abscisic alcohol by the subsequent reaction. Two intermediate metabolites, 9'-cis-neoxanthin and 9-cis-violaxanthin, have been identified in light-grown and etiolated leaves, and in roots of a variety of species [32]. Biochemical evidence has suggested the occurrence of this pathway in various eukaryotes and in archaea [33].

The existence of the sugar phosphates Glyceraldehyde-3P, Ribulose-5P, Xylulose-5P, Fructose-6P and Glucose-6P in the pentose-phosphate pathway (PPP) are found in [34-36] (Figs. 10 and 12 in Additional File 1). The major pathway for glucose-6P metabolism in *E. coli *in [37] is the same as obtained from our proposed methodology (Fig. 11 in Additional File 1).

#### A possible biological validation

Here we highlight some of the salient features of our method and try to argue that the results obtained thereof might not be a mathematical artefact. The present method maximizes the rate of production of biomass which in a way should be consistent with the law of mass action and subsumes the free energy minimization principle. Then we use the stoichiometric matrix based on these two fundamental and model independent principles. In our results, we are able to identify the true positive and the true negative scenarios correctly which could be a pointer to the fact that our method has not introduced any artefact in its formulation. As for the intermediate scenario our method for real systems so far has not produced any false positive or false negative results.

Considering the pentose phosphate pathway in the organism *E. coli *K-12 MG1655 (Fig. 10 in Additional File 1) we have found the path starting from the metabolite *α*-D-Glucose-6P to reach the target metabolite D-Glyceraldehyde-3P and D-Fructose-6P. We have observed the relative values of the components of the flux vector **v **that are involved in the aforesaid resulting pathway. The value of *v*_6 _is greater than *v*_5_, and the value of *v*_21 _is greater than *v*_22_. This leads us to obtain the target metabolite.

Starting from any intermediate metabolite, e.g., *β*-D-Glucose-6P, D-Glucono-1,5 lactone-6P and 6-Phospho-D-Gluconate, we were able to reach the target metabolite D-Glyceraldehyde-3P and D-Fructose-6P. While considering a particular intermediate metabolite, we noted the relative values of the components of the flux vector **v**. We observed that the relative values of the components of the flux vector **v **while obtaining the path from any intermediate metabolite to the target are of the same order of magnitude as that obtained by considering the original path from *α*-D-Glucose-6P to reach the target metabolite.

Moreover, we considered some other metabolite as starting substrate that are not on optimal path, and found that they did not lead to the target metabolite. For example, starting from D-Ribose-5P, we were able to reach the metabolite *PRPP *for most of the values of *λ *lying between 0.1 and 1.0, in steps of 0.1. For low values of *λ*, we could reach the metabolite D-Erythrose-4P and D-Fructose-6P. Thus we can conclude that any intermediate metabolite on optimal path produces the target metabolite, and it is independent of the starting metabolite. As desired, the metabolite not on optimal path do not lead to the target metabolite D-Glyceraldehyde-3P and D-Fructose-6P even via the reverse path. Similar observations were found for the other pathways.

We took two intermediate metabolites *β*-D-Glucose-6P and D-Glucono-1,5 lactone-6P that are indeed on the final path as identified by the present method, and we found that in most of the cases it led to the target. This shows that the method that we have developed is independent of choice of the initial substrate. Then we chose another substrate which also belongs to the path identified by us but this time the substrate could lead to various branches, of which one would eventually lead us to the target. We found that for certain range of values of the parameter *λ*, this would always lead us to the target, picking up the correct branch that is most of the time followed by the organism. Now for certain other ranges of the values of the parameter *λ*, the other branches in the pathway were picked up. In all the cases, the relative strength of the vector **v **reflects the correct strength that would drive the path from the starting substrate to the target. We have observed this feature for all the real life paths.

The kind of constraint that we have imposed on to the systems must have captured the essential biochemistry of the systems. That is why the method becomes independent of the choice of the various substrates within the conscensus pathway and makes our methodology quite a general one without centering around any specific model of the system.

## Discussion

The method developed in this article may be useful for manipulating a metabolic pathway to achieve some desired goals constituting some tasks of metabolic engineering. Here we describe a few examples where this method may be useful.

**Figure 6:**
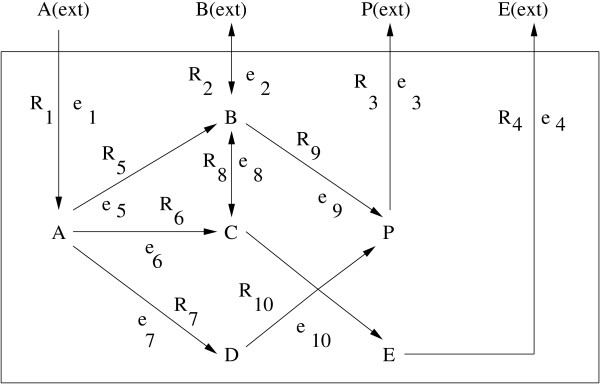
**The corresponding enzymes of the synthetic reaction system in Fig. 2**. The synthetic reaction system in Fig. 2 and their corresponding enzymes.

• Let us consider the synthetic pathway in Fig. 2 and redraw it in Fig. 6 incorporating the enzymes *e*_1_, *e*_2_,...*e*_10 _mediating the reactions *R*_1_, *R*_2_,...*R*_10_, respectively. For this system, we have already determined *R*_1 _→ *R*_5 _→ *R*_9 _→ *R*_3 _as an optimal pathway through which the amount of the product P becomes maximum. We have also found that the concentration of the enzymes *e*_1_, *e*_2_,...*e*_10 _that is required to get this optimal pathway is 0.88 for *e*_1_, 0.80 for *e*_5_, 0.80 for *e*_9 _and 0.86 for *e*_3_. For some reasons, let us say, the concentration of the enzyme *e*_5 _becomes low (~0). Under this situation, the amount of the target product will also be less. On application of the present method on this system, we would be able to identify an optimal pathway and thereby the reason behind the situation. Then we can make necessary arrangement to activate the corresponding gene and thereby leading to the formation of the enzyme in higher concentration.

• Consider the example of reducing the amount of acetate in glycolytic pathway as done by Yang et al [38]. Here the authors have proposed a method of adding a new path of forming Acetoin for reducing the amount of acetate. However, this problem may boil down to determining an optimal metabolic pathway through which the amount of acetate is minimum. Then we can apply our method to this problem for determining this optimal pathway and finally inhibit the other paths but only express this optimal path. This will lead to the formation of acetate to a minimum amount. The amount of acetate will be minimum if this optimal path is only expressed and the others are inhibited.

• For the third example, let us assume there are *n *paths *P*_1_, *P*_2_,...*P*_*n *_starting from a given substrate to yield a given target metabolite. Also assume that out of these *n *pathways, there are multiple optimal pathways *P*_1_, *P*_2_,...*P*_*m *_(*m *<*n*) through which the amount of the target metabolite becomes maximum. Now if we want to avoid a particular pathway, we may inhibit (by some means) the genes producing some of the enzymes catalyzing the reactions in that pathway.

## Conclusions

Here we have developed a simple method for identifying an optimal metabolic pathway through which a metabolite attains a maximum rate of growth on a given substrate. The method involves formulation of the rate of yield of a metabolite incorporating weighting coefficients indicating the concentration levels of enzymes catalyzing biochemical reactions in the pathway. A new constraint incorporating these weighting coefficients has been defined. Using the method, a set of flux vectors has been generated, which has then been used to determine a set of above-mentioned weighting coefficients giving rise to a maximum rate of yield of a metabolite, starting from a given substrate. The entire method is based on well known flux balancing approach.

It is to be mentioned here that the extreme pathway analysis [11] does not consider the effect of enzymes catalyzing the reactions in a metabolic pathway. On the other hand, the method developed in this article involves enzyme concentration in its formulation; thereby it is closer to the real life situations than the extreme pathway analysis. The other difference between the said two methods is that extreme pathway analysis finds the flux vectors through optimization, whereas the present method generates a subset of possible flux vectors and finds an optimal pathway in terms of weighting coefficients reflecting enzyme concentration. Moreover, the extreme pathway analysis considers individual reactions in the pathway in a sequential manner, whereas the present method considers all the reactions in parallel.

It has been observed that the method though simple enough, is able to identify the optimal pathways which conform to the results of some earlier studies. The method can suitably be used using reaction databases without going into complex mathematical calculations, and without using various kinetic parameters that are hard to be estimated. Comparative analysis of the results obtained by the present method with that of the extreme pathway analysis shows that the present method has been able to identify optimal pathways correctly for almost all the pentose phosphate and glycolytic pathways considered here. The present method has identified a carotenoid biosynthesis pathway that is closer to some earlier investigations than that obtained by the extreme pathway analysis. All the optimal real life pathways have been biologically validated. Finally, possible direct impact of the method on certain problems of metabolic engineering has been pointed out.

Here we have assumed that a large amount of substrate is present. This assumption implies that any influx of the substrate from the other pathways does not have any effect on the rate of production of the corresponding product, due to limited supply of enzymes. Moreover, for simplicity, we have not considered any feedback inhibition on the enzyme activity. In other words, we are considering only the fraction of enzyme molecules that have not been inactivated due to feedback inhibitions. Incorporation of feedback inhibitions on enzyme activity forms a scope for further investigation.

In biochemical networks, crosstalk often occurs, which deals with multiple inputs and overlapping outputs [39]. Here we are dealing with metabolic networks. If crosstalk occurs in the networks under consideration then there may be more than one disjoint sources (metabolites) from which the target or any other intermediate metabolites on the pathway under consideration are found. In this case, we have to consider all these input metabolites of the other networks while constructing the stoichiometric matrix and generating the flux vectors. However, this forms a scope for further investigation.

## Method for identification of metabolic pathways

In this section we describe the proposed method. First of all, we make some assumptions based on which we describe the method subsequently.

### Assumptions

Here we assume that a large amount of substrate is present. Thus any sudden influx of the substrate from other pathways does not effect any change of the rate of production of the corresponding product. This is due to the limited availability of the enzymes in a pathway. In other words, the ratio of enzyme concentration to substrate concentration is very low. For simplicity, we have not considered any feedback inhibition on the enzyme activity. In other words, we are considering only the fraction of enzyme molecules that have not been inactivated due to feedback inhibitions.

### System definition

A metabolic reaction network is a collection of enzymatic reactions and transport processes. A system boundary can be drawn around all these types of reactions that constitute internal fluxes operating inside the network. The system is closed to the passage of certain metabolites while others are allowed to enter and/or exit the system based on external sources and/or sinks that are operating on the network. The existence of an external source/sink on a metabolite necessitates the introduction of an exchange flux, which allows a metabolite to enter or exit the system boundary. These fluxes represent the inputs and outputs of the system.

**Figure 7:**
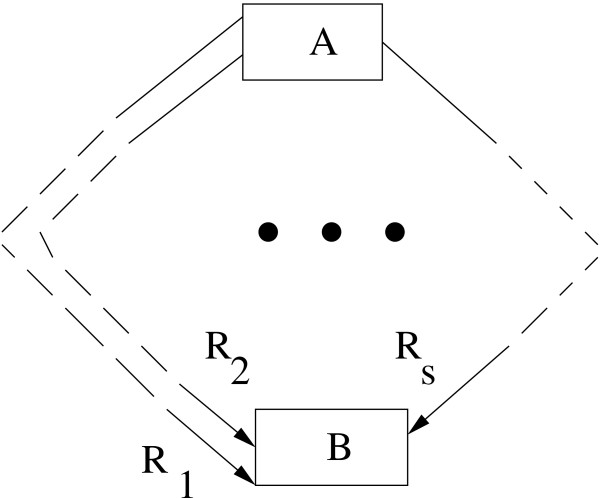
**A hypothetical biochemical reaction network**. A hypothetical biochemical reaction network.

Consider a metabolic network with the substrate (starting metabolite) A and the final metabolite B (Fig. 7). Let, the metabolite B be reached through *s *different paths. That is, there are *s *biochemical reactions/conversions *R*_1_, *R*_2_,...*R*_*s *_in the network involving the metabolite B. Let there be *n *reactions in the network, *i.e*., *n *fluxes. Some of them can be internal fluxes and the rest are exchange fluxes. If there are *p *internal and *q *exchange fluxes then *n *= *p *+ *q*. The internal and exchange fluxes are represented by *v *and *b *respectively. That is,

$$\begin{array}{ccc} v_{i} & = & v_{i},\\ & = & b_{j}, \end{array}\;\begin{array}{c} 1\leq i\leq p\\ \begin{array}{cc} p+1\leq i\leq n, & 1\leq j\leq q \end{array} \end{array}$$

Now the rate of growth of the metabolite B on the substrate A, is obtained by taking algebraic sum of the weighted fluxes of reactions *R*_1_, *R*_2_,...*R*_*s*_, and is given by

(1) $$z=\sum_{k=1}^{s}c_{k}v_{k}$$

Thus the term *z *needs to be maximized for yielding maximum rate of growth of B. Here *v*_*k *_is the flux of the reaction *R*_*k *_involving only the metabolite B [40]. The term *c*_*k *_in [0,1] denotes the weighting factor corresponding to this reaction *R*_*k*_. *c*_*k *_indicates the level of concentration of the enzyme catalyzing the reaction *R*_*k*_. *c*_*k *_= 1 indicates that the required amount of the enzyme catalyzing the reaction *R*_*k *_is present. On the other hand, *c*_*k *_→ 0 indicates that sufficient amount of enzyme is not present to carry out the reaction. Higher the value of *c*_*k*_, higher is the concentration of the enzyme and vice-versa. The term *v*_*k *_is considered to be positive if the reaction *R*_*k *_yields the metabolite B, otherwise it is negative. A reversible reaction is considered as two separate reactions corresponding to forward and backward reactions. It is to be mentioned here that the role of *c*_*i *_in Eq. (1) in extreme pathway analysis [11] is different from that in the present method. In the earlier case, **c **is a *unit vector*, along a particular flux, whereas in the present method, **c **indicates the level of concentration of the various enzymes catalyzing the reactions in the network.

### Generation of flux vectors

For solving the above-mentioned maximization problem, we require the values of the flux vectors **v **= [*v*_1_, *v*_2_,...,*v*_*n*_]^*T *^that cannot be obtained easily as the full dynamics is not known or becomes intrackable in most of the scenarios. In order to overcome this situation, we now propose an algorithm for generating flux vectors that satisfy approximately the quasi-steady state condition [11]. That is, we generate those **v **which satisfies

(2) **S.v **≈ **0**

and the inequalities in (4) and (5). Here **S **is the *m *× *n *stoichiometric matrix [41] with *m *as the number of metabolites and *n *as the number of reactions. From a reaction database, **S **can be computed. Then the flux vectors **v **form the null space of **S**. In extreme pathway analysis, the approximately equality sign in Eq. (2) is replaced by the strict equality sign as the system is in a steady state scenario. The proposed method generates the flux vectors **v **as linear combinations of the basis vectors spanning the null space of **S**, and those satisfying the inequalities (4) and (5). We cannot always guarantee (due to finite memory, and the problem of overflow or underflow for representing a numerical value) the strict equality in Eq. (2) for the flux vectors that are generated. Thus we have considered approximate equality sign instead of strict equality. Since in practical situations, *n *> *m*, Eq. (2) is under determined. So we proceed as follows:

Step I:

Generate basis vectors **v**_*b *_that form the null space of the stoichiometric matrix **S**. Let the number of such basis vectors be *l*. (This is done by using standard routines and toolboxes of MATLAB.)

Step II:

(i) Generate *l *number of positive random numbers *a*_*p*_, *p *= 1, 2,...,*l*.

(ii) Generate a vector

(3) $$v=\sum_{p=1}^{l}a_{p}v_{bp}$$

until certain inequality constraint on v is satisfied for all its components. All the internal fluxes are non-negative yielding [11]

(4) *v*_*i *_≥ 0, ∀*i*

The constraints on the exchange fluxes depends on their directions [11]. These constraints can be expressed as

(5) *α*_*j *_≤ *v*_*j *_≤ *β*_*j*_

where *α*_*j *_and *β*_*j *_are either zero, or negative and positive infinity, respectively, based on the direction of the exchange flux. The activity of these exchange fluxes is considered to be positive if the metabolite is exiting or being produced by the system, and negative if the metabolite is entering or being consumed by the system. For all metabolites in which a source or sink may be present, the exchange flux can operate in a bidirectional manner and is unconstrained. Under the existence of a source (input), *α*_*j *_is set to negative infinity and *β*_*j *_to zero. On the other hand, if only a sink (output) exists on the metabolite, *α*_*j *_is set to zero and *β*_*j *_to positive infinity. If both a source and a sink are present for the metabolite then the exchange flux is bidirectional with *α*_*j *_set to negative infinity and *β*_*j *_to positive infinity leaving the exchange flux unconstrained. For further details on these issues, one may refer to [11].

Thus we generate a large number of flux vectors, satisfying the inequality constraints, which form the data set. The flux vectors along with their corresponding weighting factors are used to determine *z*. The optimization algorithm searches through this generated data set, and estimates the values of the weighting coefficients *c*_*k *_(Eq. (7)) on convergence.

### Formulation of a new constraint

Eq. (2) describes the quasi-steady state condition, which assumes that the concentration of the enzymes catalyzing various reactions in the network is present in the system at the required level. In other words, the genes that produce these enzymes need to be expressed at the required level. For a variety of reasons, in real systems, the genes that produce these enzymes may not be expressed at the required level. This imposes restrictions on the system, and for this purpose, we define a new constraint as

(6) **S**.(**C**.**v**) = **0**

Here **C **is an *n *× *n *diagonal matrix whose diagonal elements are the components of the vector **c**. That is, if **C **= [*γ*_*ij*_]_*n *× *n*_, then *γ*_*ij *_= *δ*_*ij*_*c*_*i*_, where *δ*_*ij *_is the Kronecker delta. Note that *c*_*i *_is the weighting factor corresponding to the enzyme catalyzing ith reaction in the network, irrespective of whether the reaction involves the metabolite B or not.

Thus the problem of determining a metabolic pathway yielding maximum rate of production of a metabolite B starting from a substrate A, boils down to a maximization problem, where *z *is maximized with respect to **c**, subject to satisfying the constraint given in Eq. (6).

### Estimation of weighting coefficients *c*_*i*_

Combining Eq. (1) and Eq. (6), we can reformulate the objective function as

(7) *y *= 1/*z *+ **Λ**^*T*^.(**S**.(**C**.**v**))

that needs to be minimized with respect to the weighting factors *c*_*i *_for all *i*. The term **Λ **= [*λ*_1_, *λ*_2_,...,*λ*_*m*_]^*T *^is the regularizing parameter. For the sake of simplicity, we have considered here *λ*_1 _= ... = *λ*_*m *_= *λ *(say). Minimization of *y *can be carried out in various ways. Here we have adopted the gradient descent technique [42]. Initially, a set of random values in [0, 1] corresponding to *c*_*i*_'s are generated. The *c*_*i*_'s are then modified iteratively using the gradient descent technique, where the amount of modification for *c*_*i *_in each iteration is defined as

(8) $$\Delta c_{i}=-\eta \frac{\partial y}{\partial c_{i}}$$

The term *η *is a small positive quantity indicating the rate of modification. For computing the values of Δ*c*_*i*_'s, we use the following expression

(9) $$\Delta c_{i}=\frac{\eta }{z^{2}}\frac{\partial z}{\partial c_{i}}-\eta \frac{\partial (\Lambda^{T}.(S.(C.v)))}{\partial c_{i}}$$

Thus the modified value of *c*_*i *_is given by

*c*_*i*_(*t *+ 1) = *c*_*i*_(*t*) + Δ*c*_*i*_, ∀*i*, *t *= 0, 1, 2,...

*c*_*i*_(*t *+ 1) is the value of *c*_*i *_at iteration (*t *+ 1), which is computed based on the *c*_*i*_-value at the iteration *t*. Regularization parameter *λ *is chosen empirically. Here we are varying the value of *λ *from 0.1 to 1.0 in steps of 0.1. Using the above mentioned method, for each value of *λ*, we finally get *c*_*i*_-values for which *y *attains a minimum value. For each value of *λ*, *y *is minimized. We choose the specific *λ*-value for which the *y*-value is the minimum over all the minima obtained for different values of *λ*. The c-values corresponding to this minimum *y *are finally considered.

The vector **c **corresponds to the flux vector **v**. That is, its *i*th component *c*_*i *_(*c*_*i *_*ε *[0,1]) is associated with the flux *v*_*i *_of the *i*th reaction of a metabolic network. On minimization of *y*, some of the *c*_*i *_values will attain non-zero values in [0,1] and the others are very close to zero. We consider a metabolic path to be an optimal one, if *y *is minimum and the *c*_*i*_-values of all the enzymes catalyzing the reactions in that path are greater than zero. Otherwise, a low c-value (close to zero) corresponding to an enzyme catalyzing an intermediate reaction may result in an insufficient product. This will reduce the rate of the next reactions and hence the amount of the target metabolite. In other words, these non-zero *c*_*i*_-values indicate an optimal pathway through which the rate of yield of metabolite B, being grown on the substrate A, becomes maximum. The major differences of the method from the existing extreme pathway analysis [11] are as follows.

• Unlike the extreme pathway analysis, the present method considers the presence of enzymes.

• Extreme pathway analysis finds the flux vectors upon optimization, whereas the present method generates a set of some possible flux vectors and finds an optimal pathway in terms of weighting coefficients reflecting enzyme concentration.

• Extreme pathway analysis considers individual reactions in the pathway in a sequential manner, whereas the present method considers all the reactions in parallel.

The value of *c *corresponding to an enzyme E may be estimated *in vitro *in the following way. Let us assume the following enzymatic reactions

$$E+S{⇌}_{k_{2}}^{k_{1}}ES\to^{k_{3}}E+P$$

where S and P stand for substrate and product respectively. The terms *k*_1_, *k*_2 _and *k*_3 _are rate constants. Let us also assume that *x *mole of S can produce *y *mole of P. Under this situation, assume that [*E*_*min*_] is the minimum concentration of the enzyme E that is required to obtain the maximal rate (*V*_*max*_) of product formation. Thus an estimate of *c *may be taken as

*c *= [*E*]/[*E*_*min*_]

where [E] is the concentration level of the enzyme E which is required to get an optimal path. Note that the values of *V*_*max*_, and the rate constants can be estimated *in vitro *[43]. Thus *E*_*min *_can also be determined through [43]

*V*_*max *_= *k*_3_[*E*_*min*_]

and thereby [E] using c-value obtained by our method. On the other hand, if [E] can be determined *in vitro*, then the theoretical value of *c *(obtained by the proposed method) can be verified with the experimental value.

## Authors' contributions

RKD has conceived the study, formulated the methodology, made partial analysis of the results and has prepared the manuscript. MD has implemented the algorithms, made partial analysis of the results and has modified some parts of the manuscript. SM has made partial analysis of the results and given some fruitful suggestions while preparing the manuscript.

## Supplementary Material

Additional file 1 **Analysis of the results**. Analysis of the results for the synthetic system, pentose phosphate pathway, glycolytic pathway and the carotenoid biosynthesis pathway for different organisms. Click here for file

## References

[B1] Gadkar KG, Gunawan R, Doyle FJ (2005). Iterative approach to model identification of biological networks. BMC Bioinformatics.

[B2] Kummel A, Panke S, Heinemann M (2006). Systematic assignment of thermodynamic constraints in metabolic network models. BMC Bioinformatics.

[B3] Segre D, Vitkup D, Church GM (2002). Analysis of optimality in natural and perturbed metabolic networks. Proceedings of the National Academy of Sciences of the United States of America, PNAS.

[B4] Varma A, Palsson BO (1994). Metabolic flux balancing: basic concepts, scientific and practical use. Biotechnology.

[B5] Palsson BO (2000). The challenges of *in silico* biology. Nat Biotechnol.

[B6] Lee JM, Gianchandani EP, Papin JA (2006). Flux balance analysis in the era of metabolomics. Briefings in Bioinformatics.

[B7] Covert MW, Schilling CH, Famili I (2001). Metabolic modeling of microbial strains *in silico*. Trends Biochem Sci.

[B8] Schilling CH, Edwards JS, Palsson BO (1999). Toward metabolic phenomics: analysis of genomic data using flux balances. Biotechnol Prog.

[B9] Curto R, Sorribas A, Cascante M (1995). Comparative characterization of the fermentation pathway of *Saccharomyces cerevisiae* using biochemical systems theory and metabolic control analysis: model definition and nomenclature. Mathematical Biosciences.

[B10] Cascante M, Boros LG, Comin-Anduix B, de Atauri P, Centelles JJ, Lee PW (2002). Metabolic control analysis in drug discovery and disease. Nature Biotechnology.

[B11] Schilling CH, Letscher D, Palsson BO (2000). Theory for the systemic definition of metabolic pathways and their use in interpreting metabolic function from a pathway-oriented perspective. Journal of Theoretical Biology.

[B12] Schilling CH, Palsson BO (1998). The underlying pathway structure of biochemical reaction networks. Proceedings of the National Academy of Sciences of the United States of America, PNAS.

[B13] Klamt S, Stelling J (2003). Stoichiometric analysis of metabolic networks. Tutorial at the 4th International Conference on Systems Biology.

[B14] Kyoto encyclopedia of genes and genomes. http://www.genome.jp/kegg/pathway.html.

[B15] Covert MW, Schilling CH, Palsson BO (2001). Regulation of gene expression in flux balance models of metabolism. Journal of Theoretical Biology.

[B16] Umeno D, Arnold FH (2003). A C-35 carotenoid biosynthetic pathway. Applied and Enviromental Microbiology.

[B17] Ku B, Jeong JC, Mijts BN, Schmidt-Dannert C, Dordick JS (2005). Preparation, characterization, and optimization of an *in vitro* C30 carotenoid pathway. Applied and Enviromental Microbiology.

[B18] Naik PS, Chanemougasoundharam A, Paul Khurana SM, Kalloo G (2003). Genetic manipulation of carotenoid pathway in higher plants. Current Science.

[B19] Bendich A, Olson JA (1989). Biological actions of carotenoids. The FASEB Journal.

[B20] Sandmann G (1994). Carotenoid biosynthesis in microorganisms and plants. Eur J Biochem.

[B21] Hirschberg J, Cohen M, Harker M, Lotan T, Mann V, Pecker I (1997). Molecular genetics of the carotenoid biosynthesis pathway in plants and algae. Pure and Appl Chem.

[B22] Cunningham FX, Sun Z, Chamovitz D, Hirschberg J, Gantt E (1994). Molecular structure and enzymatic function of lycopene cyclase from the cyanobacterium synechococcus sp strain PCC7942. The Plant Cell.

[B23] Covert MW, Palsson BO (2003). Constraints-based models: regulation of gene expression reduces the steady-state solution space. Journal of Theoretical Biology.

[B24] Papin JA, Price ND, Palsson BO (2002). Extreme pathway lengths and reaction participation in genome-scale metabolic networks. Genome Research.

[B25] Santos CAF, Senalik D, Simon PW (2005). Path analysis suggests phytoene accumulation is the key step limiting the carotenoid pathway in white carrot roots. Genetics and Molecular Biology.

[B26] Grimplet J, Deluc LG, Tillett RL, Wheatley MD, Schlauch KA, Cramer GR, Cushman JC (2007). Tissue-specific mRNA expression profiling in grape berry tissues. BMC Genomics.

[B27] Davies BH (1973). Carotene biosynthesis in fungi. Pure Appl Chem.

[B28] Busch M, Seuter A, Hain R (2002). Functional analysis of the early steps of carotenoid biosynthesis in tobacco. Plant Physiology.

[B29] Armstrong GA, Hearst JE (1996). Genetics and molecular biology of carotenoid pigment biosynthesis. The Journal of the Federation of American Societies for Experimental Biology.

[B30] Saiz M, Paz B, Fuente JL, Nieto MJ, Cabri W, Barredo JL (2004). Blakeslea trispora genes for carotene biosynthesis. Applied and Environmental Microbiology.

[B31] Mcdermott JCB, Brown DJ, Britton G, Goodwin TW (1974). Alternative pathways of zeaxanthin biosynthesis in a flavobacterium species. Biochem J.

[B32] Parry AD, Horgan R (1991). Carotenoids and abscisic acid (ABA) biosynthesis in higher plants. Physiologia Plantarum.

[B33] Smit A, Mushegian A (2000). Biosynthesis of isoprenoids via mevalonate in archaea: the lost pathway. Genome Research.

[B34] Huck JH, Struys EA, Verhoeven NM, Jakobs C, van der Knaap MS (2003). Profiling of pentose phosphate pathway intermediates in blood spots by tandem mass spectrometry: application to transaldolase deficiency. Clinical Chemistry.

[B35] Verho R, Londesborough J, Penttila M, Richard P (2003). Engineering redox cofactor regeneration for improved pentose fermentation in *Saccharomyces cerevisiae*. Applied and Environmental Microbiology.

[B36] Sillero A, Selivanovy VA, Cascante M (2006). Pentose phosphate and calvin cycles: Similarities and three-dimensional views. Biochemistry and Molecular Biology Education.

[B37] El-Kazzaz W, Morita T, Tagami H, Inada T, Aiba H (2004). Metabolic block at early stages of the glycolytic pathway activates the Rcs phosphorelay system via increased synthesis of dTDP-glucose in *Escherichia coli*. Molecular Microbiology.

[B38] Yang YT, Bennett GN, San KY (1998). Genetic and metabolic engineering. Electronic Journal of Biotechnology.

[B39] Papin JA, Palsson BO (2004). Topological analysis of mass-balanced signaling networks: a framework to obtain network properties including crosstalk. Journal of Theoretical Biology.

[B40] Shlomi T, Berkman O, Ruppin E (2005). Regulatory on/off minimization of metabolic flux changes after genetic perturbations. Proceedings of the National Academy of Sciences of the United States of America, PNAS.

[B41] Urbanczik R, Wagner C (2005). Functional stoichiometric analysis of metabolic networks. Bioinformatics.

[B42] Haykin S (2001). Neural Networks.

[B43] Stryer L, Berg JM, Tymoczko JL, Clarke ND (1998). Biochemistry.

