# Supplementary material for: Incorporation of enzyme concentrations into FBA and identification of optimal metabolic pathways
Source: BMC Syst Biol. 2008 Jul 18;2:65. doi: 10.1186/1752-0509-2-65 (PMC2533768; doi:10.1186/1752-0509-2-65)
Supplement: Additional file 1 — Analysis of the results. Analysis of the results for the synthetic system, pentose phosphate pathway, glycolytic pathway and the carotenoid biosynthesis pathway for different organisms. [file 1752-0509-2-65-S1.pdf]

Additional file 1: Analysis of the results for the synthetic system, pentose phosphate pathway, glycolytic pathway and the carotenoid biosynthesis pathway for different organisms

On the system in Fig. 8

Fig. 8 shows a biochemical reaction system consisting of the 5 metabolites. Excepting B, all the other metabolites are involved in 4 exchange fluxes. There are 7 internal fluxes. Here the constraints on the 4 exchange fluxes ($b_1 - b_4$) are as follows [1]:

$$\alpha = (0, 0, 0, 0); \beta = (\infty, \infty, \infty, \infty).$$

The constraints on all the 7 internal fluxes are that these fluxes are all positive. As in the previous synthetic system in the manuscript (Fig. 2), flux vectors were generated. If we want to maximize the rate of yield of E for growth on substrate A, we have to maximize the quantity $z = c_6v_6 + c_7v_7 - c_{11}b_4$. Applying the present method, we have obtained the pathway $R_1 \rightarrow R_2 \rightarrow R_3 \rightarrow R_7 \rightarrow R_9$ as an optimal one, which is again conforming to [1]. Here R_1: Ext $\rightarrow$ A, R_2: A $\rightarrow$ B, R_3: B $\rightarrow$ C, R_4: B $\rightarrow$ D, R_5: D $\rightarrow$ B, R_6: C $\rightarrow$ D, R_7: C $\rightarrow$ E, R_8: D $\rightarrow$ E, R_9: E $\rightarrow$ Ext. It is to be mentioned that 100 iterations were required for minimizing y. The optimal pathway is obtained at $\lambda = 0.5$.

Now we intend to apply the method to a few real life pathways where there are only internal fluxes. All the internal fluxes are positive. They are more complex than the synthetic ones.

Carotenoid biosynthesis

Carotenoids are organic pigments that are naturally occurring in plants and some other photosynthetic organisms like algae, some types of fungus and some photosynthetic bacteria [3, 8]. Here they play a critical role in the photosynthetic process [9]. Carotenoids are known to belong to two classes. They also occur in some non-photosynthetic bacteria, yeasts and molds, where they carry out a protective function against damage by light and oxygen. Although, animals appear to be incapable of synthesizing carotenoids, many animals incorporate carotenoids from their diet. Within animals, carotenoids provide bright coloration, serve as antioxidants, and can be a source for vitamin A activity [10]. Carotenoids are natural fat-soluble pigments that play various biological roles. Structurally they are in the form of a polyene chain which is sometimes terminated by rings [4].

Carotenoid biosynthesis pathway in Fig. 9

Biosynthesis of carotenoids occurs in all photosynthetic organisms - bacteria, algae and plants, as well as in some non-photosynthetic bacteria and fungi. The intermediate steps in the carotenoid biosynthesis pathway were postulated several decades ago by standard biochemical analyses [3]. Carotenoid formation is a highly regulated process. Concentration and composition of leaf xanthophylls are affected by light intensity and the accumulation of specific carotenoids in chromoplasts of fruits and flowers is developmentally regulated. Variation in gene expression, most likely at the transcriptional level, is the key regulatory mechanism that controls carotenogenesis. The carotenoid biosynthesis genes (or cDNA) are functional when properly expressed in bacteria. Carotenogenic organisms can be found among heterotrophic bacteria and fungi (where some species possess this biosynthetic capacity) or among photosynthetic prokaryotes and eukaryotes. In the photosynthetic lower and higher plants carotenogenesis is obligatory for their photosynthetic activity.

The most universal carotenoid biosynthesis pathway is the sequence leading to the formation of p-carotene. The initial reaction yielding phytoene, the first carotene of the pathway, is the condensation of two molecules of geranylgeranyl diphosphate (GGPP) as an intermediate [4]. In green algae and higher plants α-carotene carrying a β- and an ε-ionone ring is formed simultaneously from lycopene. Xanthophylls are derived from α- and β-carotene by introduction of oxygen groups. The carotenoid biosynthesis pathway in Rhodobacter branches off at the stage of neurosporene. The entire carotenoid biosynthesis pathway is a part of the terpenoid metabolism with formation of prenyl diphosphates as a common sequence for chain elongations. From the different prenyl diphosphates formed, specific routes branch off into various terpenoid end products. Some investigations suggest that the dimerization of GGPP leads to phytoene as the first carotene [4]. However, there are several indications that carotenoid biosynthesis relies on its independent

synthesis of GGPP. Furthermore, during fruit ripening, which is accompanied by massive carotenoid formation in capsicum, expression of the GGPP synthase gene is strongly enhanced [4]. Consequently, the start of the specific biosynthesis pathway of carotenoids can be considered to occur with the synthesis of GGPP [4].

Carotenoids are generally synthesized as all-trans isomers or at least as a mixture in which the all-trans form is dominant. Xanthophylls are enzymatically formed oxidation products of α- and β-carotene. The latest developments demonstrated that molecular genetic approaches were of considerable help in understanding carotenoid biosynthesis [5].

In human beings, carotenoids can serve several important functions. The most widely studied and well-understood nutritional role for carotenoids is their provitamin A activity [6]. Deficiency of vitamin A is a major cause of premature death. Experimental approaches that are likely to enhance our understanding of carotenoid pathway regulation are described in [7].

Pentose Phosphate and Glycolytic pathways

Pentose Phosphate Pathway is an anabolic pathway that utilizes 6 carbons of glucose to generate NADPH and pentose (5-carbon) sugars. There are two distinct phases in the pathway. The first one is an oxidative phase, in which NADPH is generated, and the second phase is the non-oxidative synthesis of 5-carbon sugars. This pathway provides one of the three main ways the body creates reducing molecules to prevent oxidative stress, accounting for approximately 10% of NADPH production in humans.

Glycolytic pathway is a series of biochemical reactions by which a molecule of glucose is oxidized into two molecules of pyruvic acid. It is the initial process of many pathways of carbohydrate catabolism, and serves two principal functions: generation of high-energy molecules (ATP and NADH) and production of a variety of six or three-carbon intermediate metabolites that may be removed at various steps in the process for other intracellular purposes (such as nucleotide biosynthesis).

On Pentose Phosphate Pathway in *E. coli* K-12 MG1655 (Fig. 10)

We use the pentose phosphate pathway (from the KEGG database [2]) in the organism *E. coli* K-12 MG1655. Here we are maximizing the rate of yield of D-Glyceraldehyde-3P and D-Fructose-6P, starting from the substrate α-D-Glucose-6P. There are 32 fluxes and 19 metabolites (Fig. 10). We associate the weighting factors $c_1, c_2, \ldots, c_{32}$ corresponding to the enzymes catalyzing these reactions respectively. As in the previous systems, 32 dimensional flux vectors were generated. The objective function y is obtained by replacing z using $z = c_{21}v_{21} - c_{22}v_{22}$. Following the present method, we have obtained $\alpha - D - Glucose - 6P \rightarrow \beta - D - Glucose - 6P \rightarrow D - Glucono - 1, 5lactone - 6P \rightarrow 6 - Phospho - D - Gluconate \rightarrow D - Ribulose - 5P \rightarrow D - Xylulose - 5P + D - Erythrose - 4P \rightarrow D - Glyceraldehyde - 3P + D - Fructose - 6P$ as an optimal pathway. For small values of λ, it requires more or less 50 iterations for convergence. As we increase the value of λ from 0.1 to 1.0, the optimal pathway is obtained within a few iterations (less than 50). The optimal pathway is obtained at $\lambda = 0.5$.

On Glycolytic Pathway in *E. coli* K-12 MG1655 (Fig. 11)

The glycolytic pathway in *E.coli* K-12MG1655 consists of 14 metabolites and 30 fluxes (Fig. 11). The starting metabolite is α-D-Glucose-6P and the target product is pyruvate. Here we are maximizing the rate of yield $z = c_{30}v_{30}$ of pyruvate, starting from the substrate α-D-Glucose-6P. We have obtained the pathway $\alpha - D - Glucose - 6P \rightarrow \alpha - D - Glucose - 1P \rightarrow \alpha - D - Glucose \rightarrow \beta - D - Glucose \rightarrow \beta - D - Glucose - 6P \rightarrow \beta - D - Fructose - 6P \rightarrow \beta - D - Fructose - 1, 6P2 \rightarrow Glyceraldehyde - 3P \rightarrow Glycerate - 1, 3P2 \rightarrow Glycerate - 3P \rightarrow Glycerate - 2P \rightarrow Phosphoenolpyruvate \rightarrow Pyruvate$ as an optimal one. For small values of λ, it requires more or less 46 iterations for convergence. As we increase the value of λ from 0.1 to 1.0, the optimal pathway is obtained within a very few iterations. The optimal pathway is obtained at $\lambda = 0.5$.

On Pentose Phosphate Pathway in *P. falciparum* (Fig. 12)

The Pentose phosphate pathway in *P. falciparum* consists of 14 metabolites and 24 fluxes (Fig. 12). As in the previous cases, the rate of yield $z = c_{13}v_{13} - c_{14}v_{14}$ of D-Glyceraldehyde-3P is maximized starting from the substrate α-D-Glucose-6P. Following the present method, we have obtained the same optimal pathway as $\alpha - D - Glucose - 6P \rightarrow \beta - D - Glucose - 6P \rightarrow D - Glucono - 1, 5lactone - 6P \rightarrow 6 - Phospho - D - Gluconate \rightarrow D - Ribulose - 5P \rightarrow D - Xylulose - 5P \rightarrow D - Glyceraldehyde - 3P$ shown by bold (black) arrows, as in the previous real life example. Here for $\lambda = 0.7$, we got the optimal

pathway within 70 iterations.

References

1. C. H. Schilling and B. O. Palsson, "The underlying pathway structure of biochemical reaction networks," *Proceedings of the National Academy of Sciences of the United States of America, PNAS*, vol. 95, pp. 4193–4198, 1998.

2. http://www.genome.jp/kegg/pathway.html

3. D. Umeno and F. H Arnold, "A c-35 carotenoid biosynthetic pathway," *Applied and Enviromental Microbiology*, vol. 69, pp. 3573–3579, 2003.

4. G. Sandmann, "Carotenoid biosynthesis in microorganisms and plants," *Eur. J. Biochem.*, vol. 223, pp. 7–24, 1994.

5. J. Hirschberg, M. Cohen, M. Harker, T. Lotan, V. Mann, and I. Pecker, "Molecular genetics of the carotenoid biosynthesis pathway in plants and algae," *Pure and Appl. Chem*, vol. 69, pp. 2151–2158, 1997.

6. G. Britton, "Structure and properties of carotenoids in relation to function," *The Journal of the Federation of American societies for experimental biology*, vol. 9, pp. 1551–15585, 1995.

7. F. X. Cunningham, "Regulation of carotenoid synthesis and accumulation in plants," *Pure Appl. Chem.*, vol. 74, pp. 1409–1417, 2002.

8. B. Ku, J. Jeong, B. N. Mijts, C. S. Dannert, and J. S. Dordick, "Preparation, characterization, and optimization of an in vitro c30 carotenoid pathway," *Applied and Enviromental Microbiology*, vol. 71, pp. 6578–6583, 2005.

9. P.S. Naik, A. Chanemougasoundharamd, Paul Khurana S.M., and G. Kalloo, "Genetic manipulation of carotenoid pathway in higher plants," *Current Science*, vol. 85, pp. 1423–1430, 2003.

10. A. Bendich and J. A. Olson, "Biological actions of carotenoids," *The FASEB Journal*, vol. 3, pp. 1927–1932, 1989.

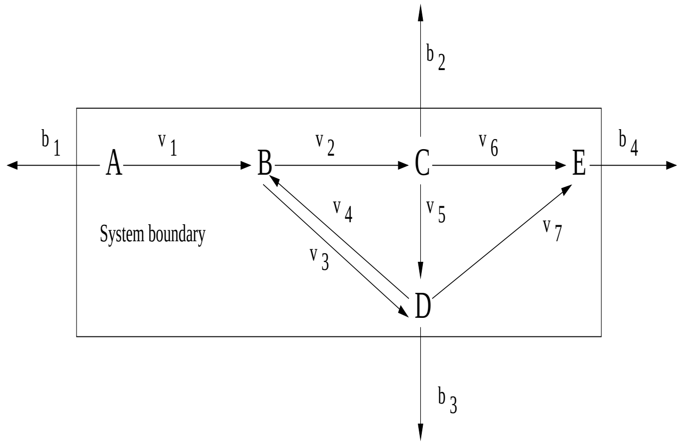

Figure 8: A chemical reaction network consisting of the 5 metabolites, 7 internal fluxes ($v_1 - v_7$) and 4 exchange fluxes ($b_1 - b_4$), giving rise to a total of 11 fluxes.

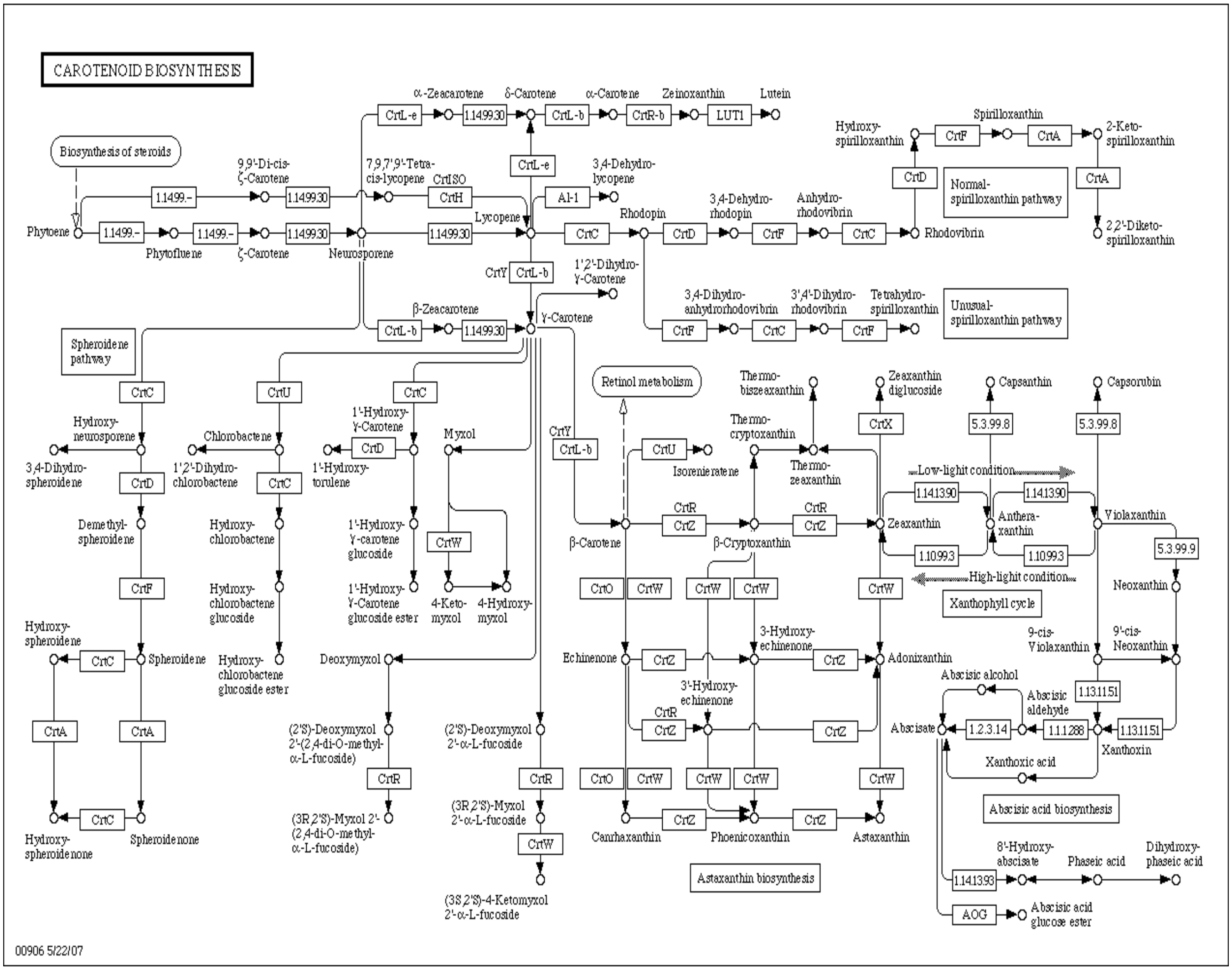

Figure 9: Carotenoid biosynthesis pathway consists of 83 metabolites and 100 fluxes (reversible reactions are shown by double arrows). The starting metabolite is phytoene and the target product is abscisic alcohol respectively.

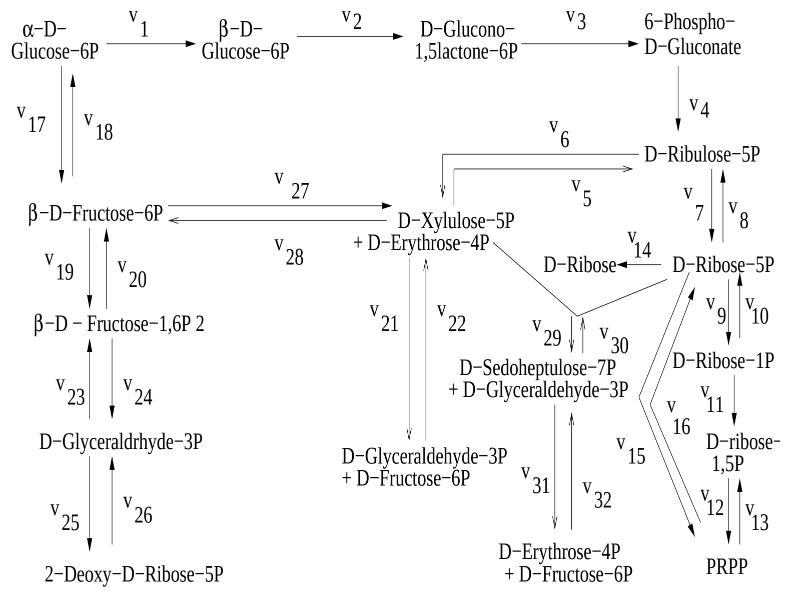

Figure 10: Pentose Phosphate pathway in *E.coli* K-12MG1655 consisting of 19 metabolites and 32 fluxes (reversible reactions are shown by double arrows). The starting metabolite is α-D-Glucose-6P and the target products are D-Glyceraldehyde-3P and D-Fructose-6P respectively.

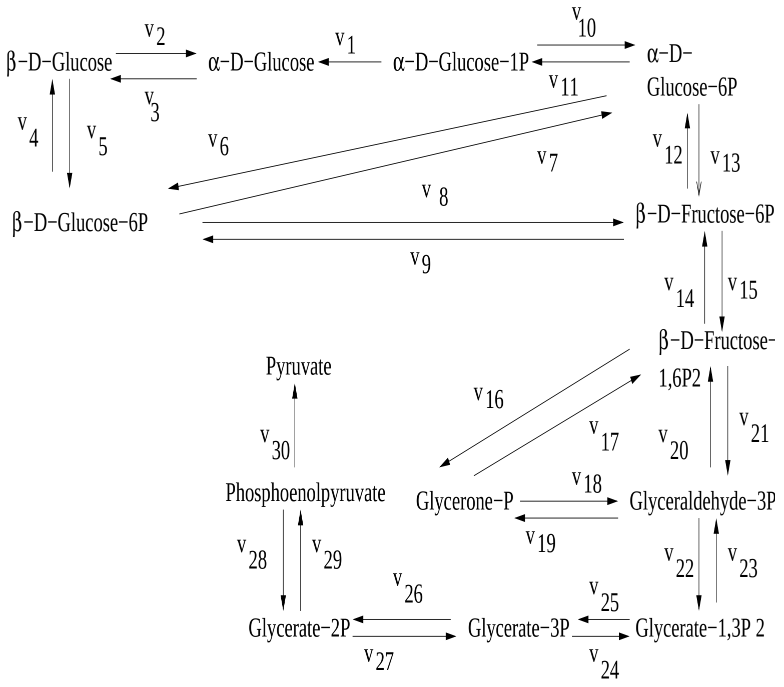

Figure 11: Glycolytic pathway in E.coli K-12MG1655 consisting of 14 metabolites and 30 fluxes (reversible reactions are shown by double arrows). The starting metabolite is α-D-Glucose-6P and the target product is pyruvate respectively.

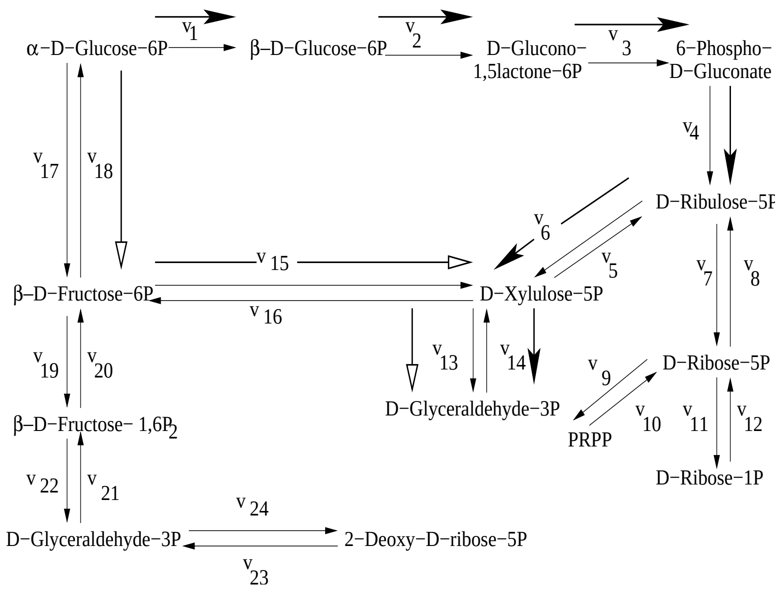

Figure 12: Pentose phosphate pathway in *P. falciparum* consists of 14 metabolites and 24 fluxes (reversible reactions are shown by double arrows). The starting metabolite is α-D-Glucose-6P and the target product is D-Glyceraldehyde-3P respectively. The bold (black) arrows represent the optimal pathway as obtained by the present method and the bold (white) arrows represent the optimal pathway as obtained by the extreme pathway analysis.
